# Poorly processed reusable surface disinfection tissue dispensers may be a source of infection

**DOI:** 10.1186/1471-2334-14-37

**Published:** 2014-01-21

**Authors:** Günter Kampf, Stina Degenhardt, Sibylle Lackner, Katrin Jesse, Heike von Baum, Christiane Ostermeyer

**Affiliations:** 1 BODE SCIENCE CENTER, Bode Chemie GmbH, Melanchthonstr. 27, 22525 Hamburg, Germany; 2 Institut für Hygiene und Umweltmedizin, Ernst-Moritz-Arndt Universität Greifswald, Walther-Rathenau-Str. 49a, 17475 Greifswald, Germany; 3 Microbiology, Bode Chemie GmbH, Melanchthonstr. 27, 22525 Hamburg, Germany; 4 Department for Medical Microbiology and Hygiene, University Hospital Ulm, Albert-Einstein-Allee 23, 89091 Ulm, Germany

**Keywords:** Surface disinfection, Reusable dispenser, Surface-active biocidal ingredients, Bacterial contamination, Achromobacter spp, Adaptation, Biofilm

## Abstract

**Background:**

Reusable surface disinfectant tissue dispensers are used in hospitals in many countries because they allow immediate access to pre-soaked tissues for targeted surface decontamination. On the other hand disinfectant solutions with some active ingredients may get contaminated and cause outbreaks. We determined the frequency of contaminated surface disinfectant solutions in reusable dispensers and the ability of isolates to multiply in different formulations.

**Methods:**

Reusable tissue dispensers with different surface disinfectants were randomly collected from healthcare facilities. Solutions were investigated for bacterial contamination. The efficacy of two surface disinfectants was determined in suspension tests against two isolated species directly from a contaminated solution or after 5 passages without selection pressure in triplicate. Freshly prepared use solutions were contaminated to determine survival of isolates.

**Results:**

66 dispensers containing disinfectant solutions with surface-active ingredients were collected in 15 healthcare facilities. 28 dispensers from nine healthcare facilities were contaminated with approximately 10^7^ cells per mL of *Achromobacter species 3* (9 hospitals), *Achromobacter xylosoxidans* or *Serratia marcescens* (1 hospital each). In none of the hospitals dispenser processing had been adequately performed. Isolates regained susceptibility to the disinfectants after five passages without selection pressure but were still able to multiply in different formulations from different manufacturers at room temperature within 7 days.

**Conclusions:**

Neglecting adequate processing of surface disinfectant dispensers has contributed to frequent and heavy contamination of use-solutions based on surface active ingredients. Tissue dispenser processing should be taken seriously in clinical practice.

## Background

The emergence of multi-resistant Gram-negative bacteria in healthcare associated infections has led to an increased awareness for prevention of transmission [1], e.g. by hand disinfection or targeted surface disinfection [2]. Especially surfaces in the immediate proximity of patients and those with frequent hand contacts should be wiped regularly with a surface disinfectant which may contain quaternary ammonium compounds (QAC), amines, glucoprotamin or amphotensides (all summarized as “surface-active ingredients”), aldehydes, alcohols or oxygen-releasing compounds [3]. In recent years reusable tissue dispensers for surface disinfection became more popular with the aim to facilitate targeted surface disinfection in areas with frequent hand contacts or in high risk areas such as intensive care units [4]. They are also recommended as one of many measures to control outbreaks, e.g. caused by *Serratia marcescens* in neonatology units [5]. Manufacturers of dispensers usually recommended how to process them before refilling with use solutions and tissue roles but compliance with the recommendation in clinical practice is unknown. We were informed in connection with an outbreak by Serratia spp. in a neonatology unit that “Pseudomonas species” at approximately 10^7^ cells per mL was detected in a single dispenser containing a surface disinfectant solution based on the QAC benzalkonium chloride (BAC) (Exner M.; personal communication). That is why we determined the frequency of contaminated surface disinfectant solutions in reusable dispensers, the ability of isolates to multiply in different formulations and their ability to form biofilms.

## Methods

### Determination of the dispenser contamination rate in healthcare facilities

Seventy dispensers or solutions from dispensers were collected randomly from various healthcare facilities as well as information on the date of last refill, type and date of last routine dispenser processing and type of disinfectant dosage (e.g. manual dosing or use of a peripheral disinfectant dosage apparatus). The focus was on surface disinfectant solutions based only on surface-active ingredients such as quaternary ammonium compounds (QAC), amphotensides, amines or glucoprotamin, but other preparations based on alcohols or aldehydes in combination with QAC were also collected. Preferably solutions in a concentration of 0.5% which are described as effective within one hour and which are recommended in risk areas (e.g. intensive care units and operating theaters) [6] and on surfaces with frequent hand contacts were collected. Each solution was tested for bacterial contamination by serial dilution in casein peptone soymeal peptone broth containing neutralizing agents (0.1% histidin, 0.1% cysteine, 0.3% lecithin, 3% Tween 80). The combination of neutralizers was validated and effective for all tested products. Aliquots of one mL were spread on casein peptone soymeal peptone agar and incubated for 72 h. Colonies were counted to calculate the number of colony-forming units (CFU) per mL. Whenever a high bacterial contamination of the solution was found the species was identified by MALDI mass spectrometry (Bruker Daltonik GmbH, Bremen, Germany). A pulsed field gel electrophoresis (PFGE) of *Achromobacter species 3* isolates was performed using Xba I as restriction enzyme [7].

### Survival of isolates in disinfectant solutions over 28 days

In order to find out if isolates are capable to multiply in different types of disinfectant solutions, we contaminated 3 dispensers per product using an aliquot of 25 mL of the bacterial suspension with a cell number adjusted to approximately 10^7^ cells per mL. Three days later tissue roles were inserted (X-Wipes; Bode Chemie GmbH, Hamburg, Germany) and use solutions (0.5%) of four surface disinfectants added (Mikrobac forte and Kohrsolin FF, Bode Chemie GmbH, Hamburg, Germany; Terralin protect, Schülke & Mayr GmbH, Norderstedt, Germany; Incidin plus, Ecolab Deutschland GmbH, Düsseldorf, Germany). Three different types of contaminants were used: the adapted isolate *Achromobacter species* 3 directly from a contaminated surface disinfectant solution, the same isolate passaged five times on casein peptone soymeal peptone agar to allow loss of adaptation, and finally the closely related species *Achromobacter xylosoxidans* as ATCC strain 27061. A dispenser was filled with 2.5 L of disinfectant solution. Dispensers with the disinfectant solution and a role of tissues were left at room temperature for 28 days. A sample of the disinfectant solution was taken on days seven, 14, 21 and 28. Serial dilution was performed in casein peptone soymeal peptone broth containing neutralizing agents (0.1% histidin, 0.1% cysteine, 0.3% lecithin, 3% Tween 80). The number of colony-forming units (CFU) per mL was determined.

### Adaptation of isolates to surface disinfectant

In order to find out if the isolates detected in surface disinfectant solutions were adapted to the formulation, the bactericidal activity of Mikrobac forte and Incidin plus (1% for 30 min, 0.5% for one h, 0.25% for four h) was determined according to EN 13727 [8] in triplicate under dirty conditions against the species that were detected in the solutions with the same strain at passage zero directly from the contaminated disinfectant solution and passage five (five passages on casein peptone soymeal peptone agar at 37°C without selection pressure). Corresponding ATCC strains were also tested. An increase of susceptibility of the isolates from passage zero to passage five was regarded as evidence for adaptation.

### Biofilm formation in disinfectant solution over 23 days

Biofilm formation was measured in triplicate with one strain of *Achromobacter species 3* and one strain of *Serratia marcescens* as described by O’Toole et al. [9]. Both strains were used in the adapted (passage 0) and de-adapted cell stage (passage 5). A microtiter plate based on polypropylene was used (Thermo Fisher Scientific, Langenselbold, Germany) which is the compound most reusable dispenser for surface disinfection tissues are made off. The contamination fluid for both species was prepared in disinfectant solution of products based on surface-active ingredients (Mikrobac forte, Bode Chemie GmbH, Hamburg, Germany; Terralin protect, Schülke & Mayr GmbH, Norderstedt, Germany; Incidin plus, Ecolab Deutschland GmbH, Düsseldorf, Germany), sterile physiological sodium chloride solution (negative control) and tryptic soy broth (TSB; positive control) resulting in a cell concentration of approximately 10^7^ CFU/mL. Microtiter plates were filled with 300 μL of contamination fluid per well and left for 3 days under a work bench for sterile goods. Each well was then filled with 300 μL of disinfectant solution (0.5%), physiological sodium chloride solution or tryptic soy broth. Microtiter plates were left at room temperature. Biofilm formation was quantified after 2, 3, 4, 5 and 24 h and after 2, 8, 10 and 23 days. At each time point plates were dumped out in order to remove any liquid, gently submerged in a tub of water, stained with 300 μL of 2% crystal violet for 15 min, submerged again in a tub of water, and finally filled with 300 μL of 30% acetic acid to solubilize crystal violet. After 15 minutes at room temperature absorbance was quantified at 550 nm using 30% acetic acid in water as the blank. Absorbance in the negative control was regarded as baseline. The OD_550 nm_ for non-specific background staining of each disinfectant solution or TSB medium was subtracted from raw values for of each disinfectant solution or TSB medium. Biofilm formation in the disinfectant solution and the positive control was calculated as the ratio between their mean OD_550 nm_ and the mean OD_550 nm_ of the negative control expressed as a relative change.

## Results

Dispensers or solutions from dispensers were obtained from 15 healthcare facilities (13 hospitals, two medical practices) in four regions of Germany. 66 of them contained surface disinfectants based only on surface-active ingredients (51 of them in a concentration of 0.5%, 11 in a concentration of 0.25% and four dispensers with an unknown disinfectant concentration). Four dispensers also contained aldehyde (solution of 0.5%) or alcohol (ready to use). The mean time between the last refill and the collection of the dispenser was 17.7 days (minimum: three days, maximum 58 days). Processing of the dispensers as recommended by the manufacturer was not performed in any of the participating healthcare facilities. A heavy contamination with 10^6^ to 10^7^ cells per mL was found in 28 of the solutions with surface-active ingredients (42.4%) whereas the disinfectants containing also aldehydes or alcohol did not reveal any relevant contamination. Whenever a contamination was detected the healthcare facility was immediately informed to allow instant removal of other dispensers. *Achromobacter species 3* was identified in dispensers of all nine healthcare facilities with contaminated solutions, from one of these *Serratia marcescens* could be isolated as well. *Achromobacter xylosoxidans* was cultivated in one dispenser. Eight isolates of *Achromobacter species 3* were available for PFGE and represented seven different strains, two isolates from one hospital were genotypically identical.

At both concentrations Incidin plus showed insufficient bactericidal activity with a mean log_10_-reduction between 0.00 and 0.09 against both dispenser isolate species from passage 0 (Table 1). Mikrobac forte revealed similar results against one dispenser isolate species (mean log_10_-reduction between 0.00 and 0.08) but was found much more effective against another (mean log_10_-reduction between 2.39 and 6.04). After five passages of the same isolates the efficacy increased mostly. Against ATCC strains both surface disinfectants were highly effective.

**Table 1:** **Mean log**_**10**_**-reduction of two surface disinfectants against un-passaged and passaged isolates from contaminated use-solutions**

**Bacterial species (origin)**	**Surface disinfectant, concentration (exposure time)**	**Passage 0**	**Passage 5**
*Achromobacter species 3* (dispenser isolate)	Mikrobac forte 1% (0.5 h)	≥ 6.04	≥ 6.80
	Mikrobac forte 0.5% (1 h)	4.61 ± 0.17	≥ 6.76
	Mikrobac forte 0.25% (4 h)	2.39 ± 0.13	4.43 ± 0.09
*Serratia marcescens* (dispenser isolate)	Mikrobac forte 1% (0.5 h)	0.08 ± 0.04*	6.82 ± 0.62
	Mikrobac forte 0.5% (1 h)	0.08 ± 0.06*	2.38 ± 0.03
	Mikrobac forte 0.25% (4 h)	0.00 ± 0.02*	< 1.68
*Achromobacter xylosoxidans* (ATCC 27061)	Mikrobac forte 1% (0.5 h)	≥ 7.35	n.a.
	Mikrobac forte 0.5% (1 h)	6.29 ± 1.02	n.a.
	Mikrobac forte 0.25% (4 h)	6.28 ± 0.16	n.a.
*Achromobacter species 3* (dispenser isolate)	Incidin plus 1% (0.5 h)	0.03 ± 0.06	4.59 ± 0.06
	Incidin plus 0.5% (1 h)	0.06 ± 0.04	2.37 ± 0.01
	Incidin plus 0.25% (4 h)	0.09 ± 0.06	< 2.27
*Achromobacter xylosoxidans* (dispenser isolate)	Incidin plus 1% (0.5 h)	0.05 ± 0.03	≥ 7.02
	Incidin plus 0.5% (1 h)	0.02 ± 0.01	5.42 ± 0.13
	Incidin plus 0.25% (4 h)	0.00 ± 0.04	4.58 ± 0.07
*Achromobacter xylosoxidans* (ATCC 27061)	Incidin plus 1% (0.5 h)	≥ 7.18	n.a.
	Incidin plus 0.5% (1 h)	≥ 7.18	n.a.
	Incidin plus 0.25% (4 h)	≥ 7.19	n.a.

*Achromobacter species 3*, *Achromobacter xylosoxidans* and *Serratia marcescens* isolated from passages 0 and 5; n = 3; dirty conditions; *passage 1 was used due to the co-contamination with *Achromobacter species 3*; n.a = not applicable.

*Achromobacter species 3* was able to multiply at room temperature in three different surface disinfectants based only on surface-active ingredients (all at 0.5%; Table 2). Multiplication was found within one to four weeks up to a cell number of 10^7^ per mL regardless of using adapted or passaged cells. No multiplication of *Achromobacter species 3* was found in a surface disinfectant containing in addition aldehyde (solution of 0.5%), or alcohol (ready to use; data not shown). *Achromobacter xylosoxidans* ATCC 27061, however, was not detected over four weeks in any of the disinfectant solutions based on surface-active ingredients.

**Table 2:** Number of CFU per mL of dispenser isolates transferred in fresh surface disinfectant solution at room temperature

**Product at a dilution to be effective in 60 minutes**	**Active ingredient(s) of undiluted product; concentration (w/w)**	**Type of cells**	**Dispenser**	**CFU/mL**			
				**Day 7**	**Day 14**	**Day 21**	**Day 28**
Mikrobac forte (0.5%)	Benzyl-C12-18-alkyldimethylammoniumchloride (19.9%), N-(3-Aminopropyl)-N-dodecylpropan-1,3-diamin (5%)	Adapted cells	1	10^6^	10^7^	n.a.	n.a.
			2	10^6^	10^7^	n.a.	n.a.
			3	10^6^	10^7^	n.a.	n.a.
		De-adapted cells	1	10^7^	10^7^	n.a.	n.a.
			2	10^7^	10^7^	n.a.	n.a.
			3	10^7^	10^7^	n.a.	n.a.
		ATCC 27061	1	0	0	0	0
			2	0	0	0	0
			3	0	0	0	0
Kohrsolin FF (0.5%)	Glutaral (5%), Benzyl-C12-18-alkyldimethylammoniumchloride (3%), Didecyldimethylammoniumchlorid (3%)	Adapted cells	1	0	0	0	0
			2	0	0	0	0
			3	0	0	0	0
		De-adapted cells	1	0	0	0	0
			2	0	0	0	0
			3	0	0	0	0
Terralin protect (0.5%)	Quaternary ammonium compound, Benzyl-C12-16-alkyl-dimethylchloride (22%), 2-Phenoxyethanol (17%), Aminoalkylglycine (0.9%)	Adapted cells	1	60	10^7^	n.a.	n.a.
			2	45	10^7^	n.a.	n.a.
			3	35	10^7^	n.a.	n.a.
		De-adapted cells	1	10^7^	10^7^	n.a.	n.a.
			2	10^7^	10^7^	n.a.	n.a.
			3	10^7^	10^7^	n.a.	n.a.
		ATCC 27061	1	0	0	0	0
			2	0	0	0	0
			3	0	0	0	0
Incidin plus (0.5%)	Glucoprotamin (26%)	Adapted cells	1	10^3^	10^7^	n.a.	n.a.
			2	10^7^	10^7^	n.a.	n.a.
			3	10^3^	10^7^	n.a.	n.a.
		De-adapted cells	1	10^7^	10^7^	n.a.	n.a.
			2	10^7^	10^7^	n.a.	n.a.
			3	10^7^	10^7^	n.a.	n.a.
		ATCC 27061	1	0	0	0	0
			2	0	0	0	0
			3	0	0	0	0

*Achromobacter species 3* (passage 0 as adapted cells, and passage 5 as de-adapted cells) or *Alcaligenes xylosoxidans* ATCC 27061 after up to 28 days in a freshly prepared disinfectant solution (2.5 l) with tissues, prepared in a fresh dispenser which was first contaminated with 25 ml of inoculum (approximately 10^7^ CFU per mL).

n.a.: not available.

**Figure 1:**
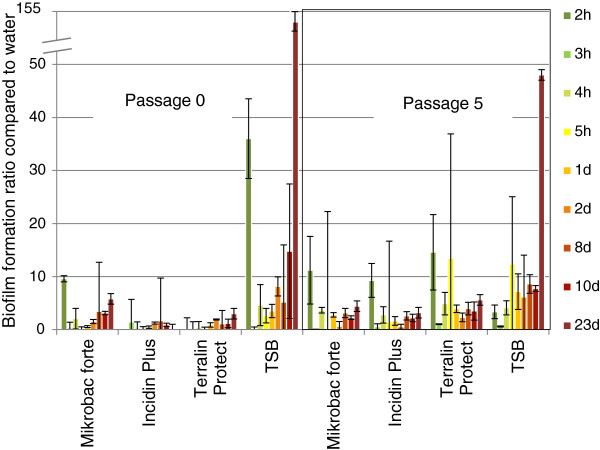
**Biofilm formation of *Achromobacter species 3 *in surface disinfectant solutions in polypropylen microtiter plates.** Cells were used as passage 0 (adapted) and passage 5 (de-adapted), surface disinfectants were prepared at 0.5%, TSB was the positive control; mean and stdev of three experiments.

**Figure 2:**
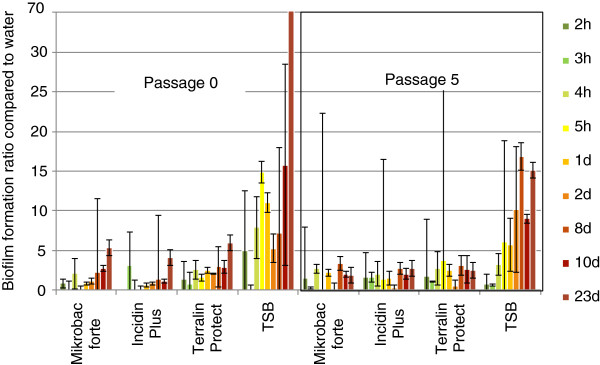
**Biofilm formation of *Serratia marcescens *in surface disinfectant solutions in polypropylen microtiter plates.** Cells were used as passage 0 (adapted) and passage 5 (de-adapted), surface disinfectants were prepared at 0.5%, TSB was the positive control; mean and stdev of three experiments.

In polypropylene microtiter plates biofilm formation was found within a few hours in all three surface disinfectant solutions contaminated with *Achromobacter species 3* or *Serratia marcescens*. After 23 days *Achromobacter species 3* formed up to five times more biofilm in all three surface disinfectant solutions in both the adapted and de-adapted cell stage (Figure 1). Similar results were found with *Serratia marcescens* (Figure 2). Both isolates were capable only in the positive control to produce large amounts of biofilm (up to 145 times) with the trend to more biofilm formation formed by the de-adapted cells. No major difference was found between the three surface disinfectant solutions.

## Discussion

The frequency of bacterial contamination found in solutions for surface disinfection based on surface active ingredients was surprisingly high with an overall rate of 42%. To the best of our knowledge none of the collected contaminated dispensers was identified as the source or associated with a nosocomial infection in the participating health care facilities although we can exclude that single transmission may have occurred but was not noted or reported. In most cases *Achromobacter species 3* were detected but *Serratia marcescens* and *Achromobacter xylosoxidans* could be isolated as well. *Achromobacter spp.* is known to cause only rarely nosocomial infections such as septicemia, pneumonia or peritonitis [10]. Especially critically ill patients e.g. from neonatology units or with immunosuppression are at risk [11]. The main cause of surface disinfectant contamination seems to be the inappropriate processing of reusable tissue dispensers. In one hospital, the dispensers were only rinsed with tap water. In another hospital the last tissue was used for a brief wipe of the dispenser’s inner surface. That is why it is essential that effective processing is not only recommended by the manufacturer but also correctly carried out by the staff of health care facilities.

Inappropriate processing has probably supported the bacterial cells to adapt to the formulations as shown with *Achromobacter species 3*, and to produce biofilm. Development of tolerance in the presence of BAC has been shown before [12]. This tolerance, however, may get lost again [12]. In our isolates *Achromobacter species 3* and *Serratia marcescens* we found the same pattern indicating that the reduced susceptibility is most likely explained by a transient adaption to BAC or glucoprotamin. Adaption to BAC has a potentially harmful consequence. It may substantially enhance biofilm production by non-BAC-resistant cells in the post-adaption period as a response to the antimicrobial stress [13]. Originally resistant isolates probably experienced lesser stress during adaptation, and hence displayed only marginal increase in biofilm formation [13]. Isolates with a resistance to BAC are also commonly resistant to different types of antibiotics [14,15] or to other types of surfactants such as benzethonium chloride or alkyldiaminoethylglycine [16]. Assuming that most isolates in clinical practice are not originally resistant, one should expect a rather high biofilm forming capacity from Gram-negative isolates surviving in or even multiplying in BAC use solution. This aspect makes a thorough cleaning of dispensers even more important not only for dispensers used over 28 days but also for dispensers used every day with a freshly prepared disinfectant solution [17].

Multiplication of *Achromobacter species 3* was found at room temperature which is not its optimum temperature for growth [18]. This could be demonstrated only in surface disinfectant solution (0.5%) but not in water indicating that at least these formulations seem to provide a “friendly environment” for bacterial growth. Different Gram-negative species are known to show different susceptibilities to BAC [19]. Solutions with specific active agents may get contaminated and thereby contribute to the transmission of pathogens – at the worst resulting in an outbreak situation [20,21]. Most outbreaks were reported with solutions based on benzalkonium chloride (BAC) which is the most commonly used QAC in surface disinfection. Whenever a contamination with BAC was detected, it was either a Gram-negative bacterial species (e.g. Pseudomonas species, *Serratia marcescens*, *Burkholderia cepacia* or *Enterobacter aerogenes*) or in two cases a mycobacterial species [20]. Multiplication of *Serratia marcescens* up to 10^7^ cells per mL has been described before in the presence of BAC or in a disinfectant based on QAC [22,23]. The reason for this, however, is not fully understood, yet, some mechanisms have been elucidated. Repeated exposure of *Serratia marcescens* to a QAC has been described to cause the emergence of mutants resistant not only to multiple species of biocides but also to structurally and functionally diverse antibiotics [24]. Exposure of the mutants to the QAC results in overexpression of an efflux pump, SdeAB [24]. Similar observations were made with glucoprotamin. Pancer et al. describe that the efficacy of glucoprotamin is reduced when microorganisms are presented in biofilm [25]. The lowest effectiveness on biofilm forming bacteria showed the disinfectant with glucoprotamine compared to formulations based on sodium dichloroisocyanurate or potassium persulfate [25]. *Alcaligenes xylosoxidans* has been described to use triclosan as a carbon source resulting in a reduction of the triclosan concentration over time [26]. In our samples from hospitals, however, we did not find a reduced level of BAC indicating that BAC is not metabolized in a relevant amount [27,28].

It becomes quite evident that the current clinical practice of dispenser processing with a “quick and dirty” approach is not suitable to ensure safe surface disinfectant solutions with formulations based on surface-active ingredients over 28 days, in some samples not even over three days. First data indicate that processing of contaminated dispensers from clinical practice is not as easy as many practitioners think if re-contamination of the disinfectant solution shall be prevented for a period of up to 28 days [29]. This finding raises serious doubts on the efficacy of manufacturer’s dispenser processing recommendations if not supported by sound scientific evidence. That is why evidence-based protocols for dispenser processing should be available as soon as possible. Some examples are available already, e.g. for effective automatic (professional washer disinfector; process for at least 5 minutes at 55°C – 60°C) and manual processes (thorough rinse with hot water, drying, thorough disinfection with alcohol-based surface disinfectant, allow to dry) [29]. These processing protocols should be accompanied with excellent staff training and stipulation of a HACCP-like quality assurance system [4] since potential errors by users may have an immediate impact. If clinical practice does not change in that respect we have to be aware that outbreaks propagated by contaminated surface disinfectant solutions based on BAC or glucoprotamine as a point source might be reported soon. Additional concern may arise because *Achromobacter xylosoxidans* is apparently a reservoir of horizontal genetic transfer elements commonly involved in spreading antibiotic resistance [30]. Inappropriately processed dispensers will allow this pathogen to persist in the environment of healthcare facilities with all possible implications.

## Conclusions

Disinfectant solutions based on surface-active ingredients from poorly processed tissue dispensers are frequently contaminated with adapted biofilm-forming Gram-negative bacteria. Effective processing of tissue dispensers is essential to eliminate them as a possible source of pathogen transmission and consecutive infections especially if disinfectants based on QAC, amines or glucoprotamin are used.

## Abbreviations

ATCC: American type culture collection; BAC: Benzalkonium chloride; CFU: Colony-forming unit; EN: European norm; OD: Optical density; QAC: Quaternary ammonium compound; TSA: Tryptic soy agar; TSB: Trypic soy broth.

## Competing interests

GK, SD, SL, KJ and CO are paid employees of Bode Chemie GmbH, Hamburg, Germany.

## Authors’ contributions

GK and CO designed the study, SD and SL performed and analyzed all experiments except on biofilm, KJ performed and analyzed the experiments on biofilm and prepared the figures, GK made the literature search, HB organized and supervised the experiments on PFGE, GK analyzed the data and wrote the manuscript, all authors read and approved the final manuscript.

## Pre-publication history

The pre-publication history for this paper can be accessed here:

http://www.biomedcentral.com/1471-2334/14/37/prepub
